# Impact of pathogen co-detection on disease severity and clinical outcomes in children with *Mycoplasma pneumoniae* pneumonia

**DOI:** 10.1007/s12519-026-01053-2

**Published:** 2026-06-11

**Authors:** Feng Wang, Ying-Wen Wang, Jia-Yu Wang, Li-Bo Wang, Wen He, Qing Wang, Yuan-Yuan Qi, Xiao-Bo Zhang

**Affiliations:** 1 Department of Respiratory Medicine, Children’s Hospital of Fudan University, National Children’s Medical Center, Shanghai 201102, China; 2 Department of Nursing, Children’s Hospital of Fudan University, National Children’s Medical Center, Shanghai 201102, China; 3 National Health Commission Key Laboratory of Neonatal Diseases (Fudan University), Children’s Hospital of Fudan University, National Children’s Medical Center, Shanghai 201102, China

**Keywords:** Children, Co-detection, *Mycoplasma pneumoniae*, Pneumonia, Severe *Mycoplasma pneumoniae* pneumonia

## Abstract

**Background:**

Although *Mycoplasma pneumoniae* pneumonia (MPP) is a leading cause of pediatric community-acquired pneumonia, the specific clinical impact of respiratory pathogen co-detection remains incompletely understood.

**Methods:**

In this retrospective cohort study, we analyzed data from 3081 children hospitalized with confirmed MPP at a single center in China (January 2023–December 2024). Based on comprehensive respiratory pathogen testing, patients were classified into MPP mono-infection (*n* = 1173) or MPP co-detection (*n* = 1908) groups. Severe MPP (SMPP) was defined as a composite outcome per national guidelines. The primary outcome was the incidence of SMPP; secondary outcomes included specific complications, healthcare utilization, and costs. Stratified analysis by co-detection pattern (single virus, single bacterium, viral–bacterial, and multiple viruses) was performed. Multivariable logistic regression was used to assess the independent association of co-detection with SMPP, adjusting for sex, age, platelet count, C-reactive protein (CRP) level, and D-dimer level.

**Results:**

The co-detection rate was 61.9% (1908/3081). Among co-detected cases, single-viral detection was most common (41.8%), followed by viral–bacterial detection (19.1%). Adenovirus (33.5%) and rhinovirus (32.1%) were the predominant single viruses. The co-detection group exhibited a significantly higher incidence of SMPP (51.8% vs. 47.3%, *P* = 0.018), higher total hospitalization costs, longer hospital stays, and longer durations of cough and fever (all *P* < 0.001). Stratified analysis revealed that the viral–bacterial co-detection subtype consistently exhibited the most severe outcomes. After adjusting for sex, age, platelet count, CRP level, and D-dimer level, pathogen co-detection was an independent risk factor for SMPP [adjusted odds ratio (OR) = 1.31; 95% confidence interval = 1.11–1.56; *P* = 0.002]. Elevated D-dimer levels (adjusted OR = 3.66) and older age were also significant independent predictors.

**Conclusions:**

Respiratory pathogen co-detection is prevalent in children with MPP and is independently associated with disease progression to SMPP (as defined by guideline criteria), prolonged hospitalization, and increased healthcare costs. The viral–bacterial co-detection pattern is associated with the greatest risk. These findings underscore the importance of comprehensive pathogen screening in the management of pediatric MPP.

**Graphical abstract:**

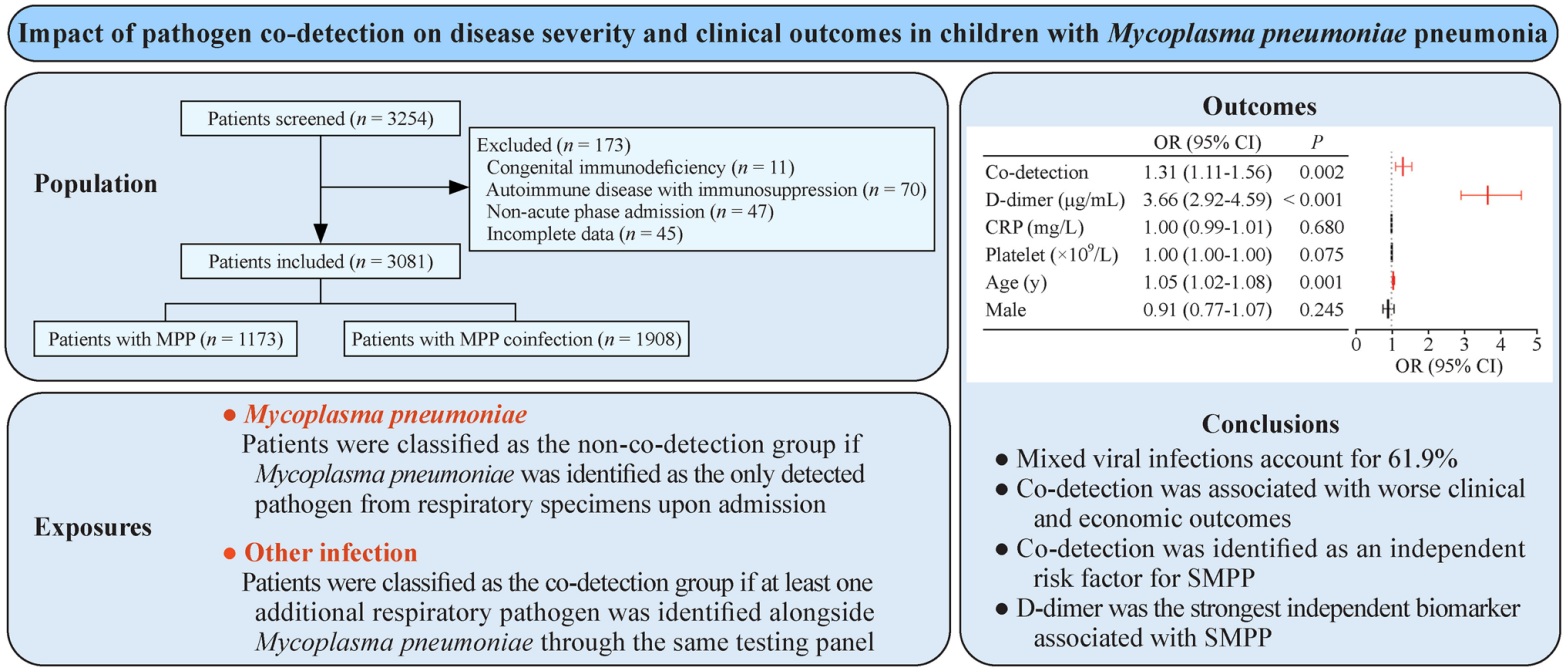

## Introduction

*Mycoplasma pneumoniae* is a leading cause of community-acquired pneumonia in children and adolescents worldwide and is responsible for a substantial proportion of both endemic cases and periodic epidemics [1, 2]. The clinical presentation of *M. pneumoniae* pneumonia (MPP) varies widely, ranging from mild, self-limiting respiratory illness to severe, refractory pneumonia requiring hospitalization, with a subset of patients developing extensive pulmonary infiltrates or severe extrapulmonary complications [3, 4]. The widespread adoption of molecular diagnostic techniques has significantly improved detection of *M. pneumoniae*, leading to more frequent and accurate diagnoses [5] and revealing that the co-detection of multiple pathogens is a common phenomenon in pediatric community-acquired pneumonia [6, 7], with viral pathogens such as adenovirus, rhinovirus, and respiratory syncytial virus frequently identified alongside *M. pneumoniae* [8], challenging the traditional paradigm of single-pathogen etiology. However, the clinical significance of respiratory co-detection in children with MPP remains unclear and is a subject of debate. Although some studies have suggested that co-detection potentiates disease severity and leads to more complicated clinical courses [9–11], others have reported minimal additional impact or even contradictory findings [12].

This ongoing uncertainty stems from methodological limitations in existing literature, including small sample sizes, inconsistent definitions of co-detection and disease severity, and inadequate adjustment for potential confounding factors. Consequently, few large-scale, comprehensive studies have systematically evaluated the clinical and economic impacts associated with MPP co-detection. Crucial questions remain: whether co-detection independently predicts progression to severe MPP (SMPP) and whether it prolongs illness duration, increases complication rates, or increases healthcare costs. Addressing these questions is essential for advancing risk stratification, optimizing clinical management decisions, and ensuring efficient resource allocation.

We conducted a large-scale retrospective cohort study at a major pediatric tertiary center. Our primary objectives were: (1) to describe the prevalence and pathogen spectrum of respiratory co-detection in a large cohort of hospitalized children with confirmed MPP; (2) to compare disease severity, clinical course, complications, and hospital resource utilization between children with MPP alone and those with MPP co-detection; and (3) to determine whether co-detection is an independent risk factor for SMPP after adjustment for key demographic and laboratory factors.

## Methods

### Setting and participants

This retrospective study enrolled children hospitalized for MPP between January 1, 2023, and December 31, 2024 at a national children’s medical center that provides care to a geographically diverse population across East China. Pediatric patients with confirmed community-acquired MPP, diagnosed through polymerase chain reaction (PCR) and serological validation (defined as a single *M. pneumoniae*-immunoglobulin M titer ≥ 1:160) in accordance with the “Diagnosis and treatment guidelines for *Mycoplasma pneumoniae* pneumonia in children (2025 edition)” [13] (ICD number: J15.700), were eligible for inclusion. SMPP was defined according to the 2025 national guidelines as a composite outcome indicating a severe disease phenotype. Any of the following manifestations indicate SMPP: (1) persistent high fever (≥ 39 °C) for ≥ 5 days or fever for ≥ 7 days with no decrease in peak temperature or subsequent increase, resulting in high fever; (2) the presence of one or more of the following: wheezing, shortness of breath, dyspnea, chest pain, or hemoptysis; (3) the development of extrapulmonary complications not meeting critical illness criteria; (4) a resting oxygen saturation ≤ 93% at sea level breathing room air; (5) imaging findings meeting any of the following: 1) high-density consolidation involving approximately 2/3 or more of one lobe, or high-density consolidation in two or more lobes (regardless of extent); 2) unilateral or bilateral diffuse bronchiolitis, possibly with bronchitis; (6) progressive worsening of clinical symptoms with imaging showing disease progression exceeding 50% within 24-48 hours; and (7) significant elevation in any the levels of C-reactive protein (CRP), lactate dehydrogenase (LDH), or D-dimer. Exclusion criteria were applied to minimize confounding: patients with underlying comorbidities (e.g., congenital immunodeficiency, malignancy, human immunodeficiency virus, tuberculosis), nonacute-phase admissions (symptom-to-admission interval > 4 weeks or radiographic improvement at admission), conditions interfering with therapeutic assessment (e.g., epilepsy), or incomplete clinical records. Ethical approval was obtained from the Research Ethics Board of Children’s Hospital (approval number: [2022] 307A), and informed consent was waived because of the retrospective nature of the study.

### Data source

Data were extracted from electronic health records (EHRs) including demographic characteristics (sex, age), symptomatic presentations (duration of fever and cough, peak body temperature), laboratory parameters (white blood cell count, neutrophil percentage, CRP level, D-dimer level, LDH level, liver function tests), microbiological analyses (macrolide resistance gene mutations, respiratory pathogen panels, sputum cultures), radiological imaging (chest computed tomography and/or posteroanterior X-ray), bronchoscopy records, and hospital resource utilization (admission/discharge dates, hospitalization costs). These multidomain data were systematically compiled to ensure robust evaluation of clinical outcomes.

### Exposures

The primary exposure was respiratory pathogen co-detection in patients with confirmed MPP. At our institution, comprehensive pathogen screening using sputum or bronchoalveolar lavage fluid samples is a standard procedure for all patients admitted with respiratory tract infections and is performed upon admission. The routine testing panel includes multiplex PCR assays for common respiratory viruses and bacterial cultures for typical and atypical pathogens. Based on microbiological findings, the non-co-detection group consisted of patients in whom MPP was the only pathogen detected, although the co-detection group included patients who had at least one additional respiratory pathogen identified alongside *M. pneumoniae*.

### Outcomes

Data for all primary and secondary outcomes were extracted from the institution’s EHRs. The primary outcomes of this study were disease severity and the incidence of in-hospital complications. Severity was graded according to the criteria predefined above. Complications were categorized as follows: (1) pulmonary complications including plastic bronchitis, pulmonary embolism, pleural effusion, necrotizing pneumonia, and acute asthma exacerbations; and (2) extrapulmonary complications, encompassing skin manifestations, hepatic impairment, renal dysfunction, cardiac involvement, hematologic abnormalities, and neurological sequelae. The secondary outcomes included: (1) total hospitalization costs (accumulated until discharge); (2) length of hospital stay (from admission to discharge); (3) duration of cough and fever (defined as the number of days from onset to complete resolution of the symptom); (4) need for bronchoscopy and multiple bronchoscopies (during hospitalization); and (5) need for respiratory support (during hospitalization), encompassing noninvasive ventilation, high-flow nasal cannula oxygen therapy, or invasive mechanical ventilation.

### Statistical analysis

Statistical analyses were performed using SPSS 26.0 (IBM). Continuous variables were assessed for normality using the Shapiro–Wilk test, with normally distributed data reported as the means ± standard deviations and compared via independent *t* tests, although non-normally distributed data were expressed as the medians (interquartile ranges) and analyzed using the Mann–Whitney *U* test. Categorical variables are presented as frequencies and percentages [*n* (%)] and were compared using Pearson’s Chi-square test or Fisher’s exact test for sparse data. Furthermore, in response to the potential heterogeneity within the co-detection group, we performed a secondary, stratified analysis. Patients with MPP co-detection were subclassified into four distinct patterns: (1) co-detection with a single virus; (2) co-detection with a single bacterium; (3) co-detection with both viruses and bacteria; and (4) co-detection with multiple viruses. Clinical outcomes were compared across these four subtypes and the MPP alone group using the Kruskal–Wallis test (for continuous variables) or the Chi-square test (for categorical variables). To evaluate the independent association between co-detection and SMPP, we performed multivariable logistic regression analysis. The model was adjusted for potential confounders including sex, age, platelet count, and CRP and D-dimer levels. To assess the potential impact of criterion contamination (as CRP and D-dimer levels are part of the SMPP definition), a sensitivity analysis was performed using a logistic regression model adjusted only for sex, age, and platelet count, excluding CRP and D-dimer levels. Missing laboratory test or imaging parameter data were handled by multiple imputation methods. Statistical significance was defined as a two-tailed *P* < 0.05.

## Results

### Study participants

**Fig. 1:**
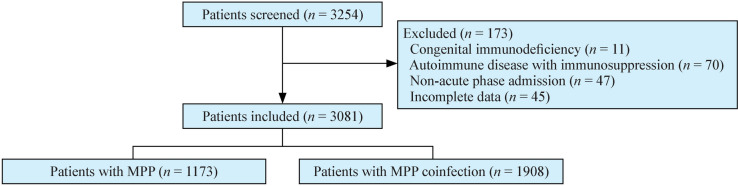
Patient selection and grouping flow diagram. *MPP Mycoplasma pneumoniae* pneumonia

Between January 2023 and December 2024, a total of 3254 pediatric patients with suspected MPP were recruited. A total of 173 patients were excluded for the following reasons: congenital immunodeficiency (*n* = 11), autoimmune disease requiring immunosuppression (*n* = 70), nonacute-phase admission (*n* = 47), or incomplete clinical data (*n* = 45). Consequently, 3081 patients were included in the final analysis. Based on comprehensive etiological testing, the cohort was stratified into two groups: 1173 (38.1%) patients with MPP alone and 1908 (61.9%) patients with MPP co-detection (Fig. 1).

### Baseline characteristics and clinical features of co-detection patients

The baseline characteristics and clinical features of the 1173 patients with MPP alone and the 1908 patients with MPP co-detection are summarized in Table 1. The two groups were comparable in terms of sex distribution (*P* = 0.941). However, the co-detection patients were significantly younger (*P* < 0.001). The prevalence of primary clinical symptoms, including cough (> 99.5% in both groups) and fever (> 94% in both groups), as well as peak body temperature, did not differ significantly between the groups (all *P* > 0.05). In terms of laboratory parameters, the co-detection group exhibited significantly higher platelet counts (*P* = 0.008) and D-dimer levels (*P* < 0.001) but lower CRP levels (*P* = 0.003). No significant intergroup differences were observed in white blood cell count, liver enzyme (alanine aminotransferase, aspartate aminotransferase) levels, lactate dehydrogenase levels, drug resistance gene detection rate, or radiological findings of pulmonary consolidation and pleural effusion (all *P* > 0.05).

**Table 1:** Comparison of general data and clinical characteristics between the two groups

Characteristics	MPP alone (*n* = 1173)	MPP co-detection (*n* = 1908)	*P*
Demographics			
Male	581 (49.5)	942 (49.4)	0.941
Age (y)	6.9 (4.9–8.7)	6.2 (4.0–8.1)	< 0.001
Symptoms			
Cough	1169 (99.7)	1900 (99.6)	> 0.999
Fever	1118 (95.3)	1797 (94.2)	0.182
Highest temperature	39.3 (39.0–40.0)	39.3 (38.9–40.0)	0.242
Laboratory findings			
WBC (× 10⁹/L)	8.0 (6.0–10.8)	8.3 (6.3–10.7)	0.110
Platelet (× 10⁹/L)	365.0 (291.0–445.0)	378.0 (305.0–453.0)	0.008
CRP (mg/L)	5.8 (2.0–11.6)	4.7 (1.6–10.0)	0.003
D-dimer (μg/mL)	0.4 (0.3–0.7)	0.4 (0.2–0.7)	< 0.001
ALT (U/L)	15.3 (12.2–20.9)	15.1 (12.2–20.7)	0.691
AST (U/L)	28.3 (23.6–34.2)	28.5 (23.8–34.6)	0.280
LDH (U/L)	321.0 (277.0–372.5)	320.0 (280.0–373.0)	0.707
Drug resistance gene	845 (72.0)	1430 (74.9)	0.076
Imaging			
Pulmonary consolidation	628 (53.5)	953 (49.9)	0.054
Pleural effusion	125 (10.7)	197 (10.3)	0.808

Data are presented as *n* (%) or median (interquartile range). *MPP Mycoplasma pneumoniae* pneumonia, *WBC* white blood cell, *CRP* C-reactive protein, *ALT* alanine aminotransferase, *AST* aspartate aminotransferase, *LDH* lactate dehydrogenase

### Pathogen spectrum and distribution among the patients with MPP co-detection

**Fig. 2:**
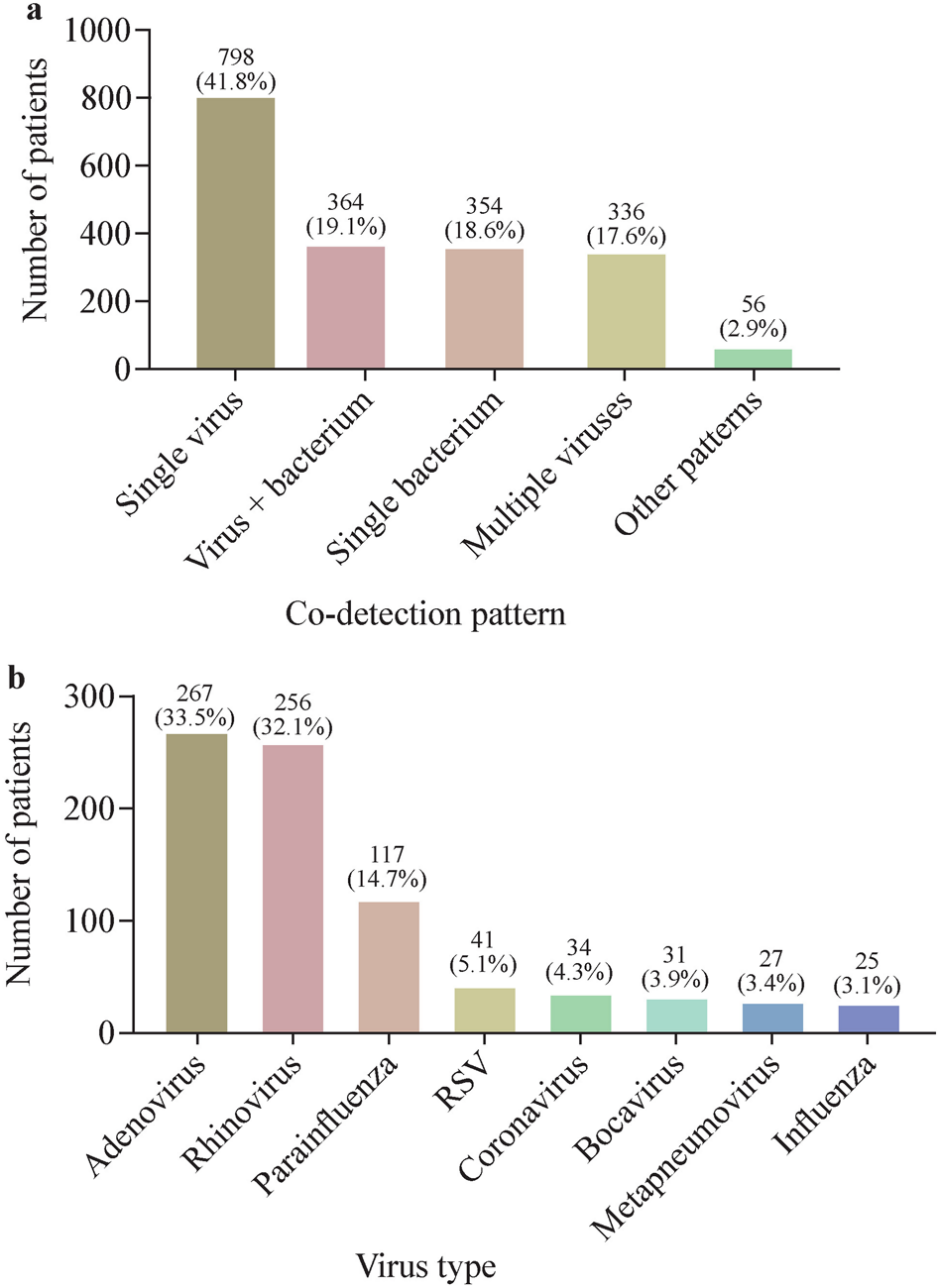
Pathogen composition in 1908 patients with *Mycoplasma pneumoniae* pneumonia co-detection. **a** Distribution of pathogen categories; **b** distribution of specific viruses among the 798 single-viral infections. *RSV* respiratory syncytial virus

Among the 1908 patients with MPP co-detection, single-viral co-detection was the most common pattern (798/1908, 41.8%), followed by viral–bacterial co-detection (364, 19.1%), single-bacterial co-detection (354, 18.6%), and co-detection with multiple viruses (336, 17.6%) (Fig. 2a). Other co-detection patterns collectively accounted for 2.9% (56/1908) of the cases. Among the 798 patients with single viruses detected, adenovirus (267, 33.5%) and rhinovirus (256, 32.1%) were the predominant viruses, followed by parainfluenza virus (117, 14.7%) and respiratory syncytial virus (41, 5.1%). Coronavirus, bocavirus, metapneumovirus, and influenza virus were identified in 4.3%, 3.9%, 3.4%, and 3.1% of these cases, respectively (Fig. 2b).

### Independent risk factors for severe *Mycoplasma pneumoniae* pneumonia

#### Univariate analysis of clinical outcomes

Clinical outcomes differed significantly between mono-infection and co-detection groups (Table 2). In terms of the primary outcomes, the co-detection group had a significantly greater incidence of SMPP (*P* = 0.018). However, the rates of overall pulmonary complications (*P* = 0.655) and extrapulmonary complications (*P* = 0.791) were comparable between the two groups. With respect to secondary outcomes, the co-detection group incurred significantly higher total hospitalization costs (*P* < 0.001), experienced longer durations of cough (*P* < 0.001) and fever (*P* < 0.001), and had a longer hospital stay (*P* < 0.001). Furthermore, compared with mono-infection group, patients with co-detection had higher rates of both bronchoscopy (*P* = 0.008) and multiple bronchoscopies (*P* = 0.024). The need for respiratory support was similar between the groups (*P* = 0.359).

**Table 2:** Comparison of clinical outcomes between children with MPP alone and those with MPP co-detection

Characteristics	MPP (*n* = 1173)	MPP co-detection (*n* = 1908)	*P*
Primary outcomes			
SMPP	555 (47.3)	988 (51.8)	0.018
Pulmonary complications	144 (12.3)	245 (12.8)	0.655
Extrapulmonary complications	98 (8.4)	166 (8.7)	0.791
Secondary outcomes			
Total cost (¥)	9161 (7365–11,269)	10,201 (8141–11,942)	< 0.001
Hospital days	4.0 (3.0–5.0)	4.0 (3.0–6.0)	< 0.001
Duration of cough (d)	8.0 (6.0–11.0)	9.0 (6.0–12.0)	< 0.001
Duration of fever (d)	6.0 (4.0–8.0)	6.0 (4.0–8.0)	< 0.001
Bronchoscopy	737 (62.8)	1289 (67.6)	0.008
Multiple bronchoscopies	103 (8.8)	216 (11.3)	0.024
Respiratory support	36 (3.1)	47 (2.5)	0.359

Data are presented as *n* (%) or median (interquartile range). *MPP Mycoplasma pneumoniae* pneumonia, *SMPP* severe *Mycoplasma pneumoniae* pneumonia

#### Analysis of clinical outcomes stratified by co-detection pattern

Given the potential heterogeneity within the co-detection group, we further stratified these patients into four distinct patterns: single-viral co-detection, single-bacterial co-detection, viral–bacterial co-detection, and multiple viral co-detections. As shown in Table 3, significant differences were observed among the five groups (MPP alone and the four co-detection subtypes) for most clinical outcomes.

**Table 3:** Comparison of clinical outcomes among children with MPP alone and different pathogen co-detection patterns

Characteristics		MPP (*n* = 1173)	Single virus co-detection (*n* = 798)	Single bacterium co-detection (*n* = 354)	Virus bacterium co-detection (*n* = 364)		Multiple viruses co-detection (*n* = 336)	*χ*^2^	*P*
Primary outcomes									
SMPP		555 (47.3)	407 (51.0)	173 (48.9)	202 (55.5)		182 (54.2)	10.558	0.032
Pulmonary complications		144 (12.3)	94 (11.8)	39 (11.0)	66 (18.1)		39 (11.6)	11.796	0.019
Extrapulmonary complications		98 (8.4)	67 (8.4)	33 (9.3)	40 (11.0)		23 (6.8)	4.310	0.366
Secondary outcomes									
Total cost (¥)		9161 (7365–11,269)	10,010 (8016–11,689)	9341 (7723–11,321)	10,662 (8625–12,754)		10,658 (8844–12,675)	89.090	< 0.001
Hospital days		4.0 (3.0–5.0)	4.0 (3.0–6.0)	4.0 (3.0–5.0)	5.0 (4.0–6.0)		5.0 (4.0–6.0)	41.207	< 0.001
Duration of cough (d)		8.0 (6.0–11.0)	8.0 (6.0–12.0)	8.0 (6.0–11.0)	10.0 (7.0–13.0)		9.0 (7.0–14.0)	31.335	< 0.001
Duration of fever (d)		6.0 (4.0–8.0)	5.0 (3.0–7.0)	6.0 (5.0–8.0)	6.0 (3.0–8.0)		6.0 (3.0–8.0)	19.949	< 0.001
Bronchoscopy		737 (62.8)	527 (66.0)	220 (62.1)	257 (70.6)		243 (72.3)	16.689	0.002
Multiple bronchoscopies		103 (8.8)	86 (10.8)	30 (8.5)	56 (15.4)		35 (10.4)	14.650	0.005
Respiratory support		36 (3.1)	17 (2.1)	4 (1.1)	15 (4.1)		9 (2.7)	7.770	0.100

Data are presented as *n* (%) or median (interquartile range). *MPP Mycoplasma pneumoniae* pneumonia, *SMPP* severe *Mycoplasma pneumoniae* pneumonia

Regarding primary outcomes, the incidence of SMPP differed significantly across groups (*P* = 0.032), with the viral–bacterial co-detection subtype exhibiting the highest rate (55.5%), followed by multiple viral co-detection (54.2%). Similarly, pulmonary complications showed significant intergroup variation (*P* = 0.019), with the viral–bacterial co-detection subtype again demonstrating the highest incidence (18.1%). In contrast, the incidence of extrapulmonary complications was comparable among all groups (*P* = 0.366).

In terms of secondary outcomes, the five groups differed significantly in total hospitalization costs (*P* < 0.001), hospital stay length (*P* < 0.001), duration of cough (*P* < 0.001), duration of fever (*P* < 0.001), bronchoscopy utilization (*P* = 0.002), and multiple bronchoscopies (*P* = 0.005). Notably, the viral–bacterial co-detection subtype consistently had the most severe disease profile, with the longest median hospital stay (5.0 days), highest median total cost (¥10,662), and highest rate of bronchoscopy utilization (70.6%). The requirement for respiratory support was similar across all groups (*P* = 0.100).

#### Multivariable analysis of independent risk factors

**Fig. 3:**
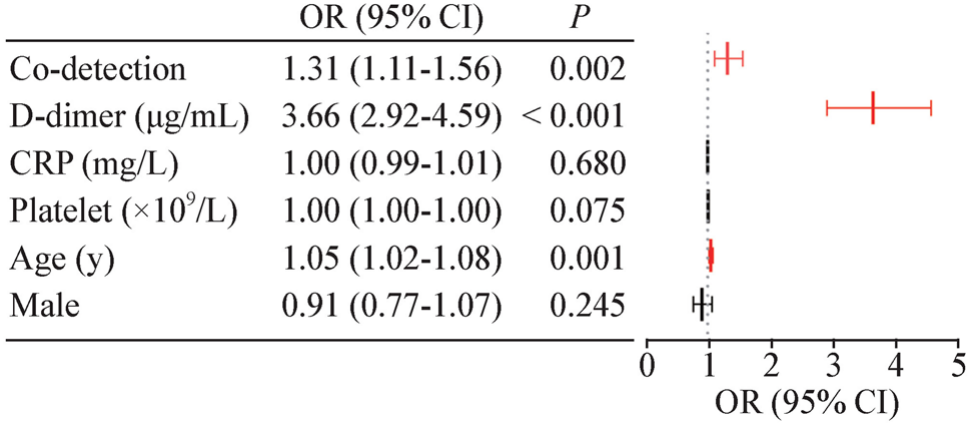
Forest plot of factors associated with severe *Mycoplasma pneumoniae* pneumonia. *CRP* C-reactive protein, *OR* odds ratio, *CI* confidence interval

Multivariable logistic regression analysis, adjusted for sex, age, platelet count, and CRP and D-dimer levels was performed to identify independent risk factors for SMPP. As shown in Fig. 3, co-detection was a significant independent predictor, with an odds ratio (OR) of 1.31 [95% confidence interval (CI) = 1.11–1.56; *P* = 0.002]. In a sensitivity analysis that excluded CRP and D-dimer levels from the multivariable model, co-detection remained independently associated with SMPP (OR = 1.22; 95% CI = 1.05–1.42; *P* = 0.008). In further sensitivity analysis adjusting for the presence of macrolide resistance genes in addition to the primary covariates, co-detection remained independently associated with SMPP (OR = 1.21; 95% CI = 1.04–1.40; *P* = 0.015). Among the analyzed covariates, D-dimer level demonstrated the strongest association with SMPP (OR = 3.66; 95% CI = 2.92–4.59; *P* < 0.001). Older age was independently associated with an increased risk of severe disease (OR = 1.05 per year increase; 95% CI = 1.02–1.08; *P* = 0.001). Platelet count showed a nonsignificant positive trend (*P* = 0.075), although CRP level (*P* = 0.680) and male sex (*P* = 0.245) were not significantly associated with SMPP in the adjusted model.

## Discussion

Our large-scale, retrospective study of more than 3000 hospitalized children with MPP provides substantial evidence that respiratory co-detection is not only highly prevalent (61.9%) but also carries significant clinical and economic implications. The key findings are threefold. First, co-detection, particularly with viral pathogens such as adenovirus and rhinovirus, was common. Second, children with MPP co-detection experienced greater disease severity, as evidenced by a higher incidence of SMPP, longer durations of fever and cough, prolonged hospitalization, and increased healthcare costs. Third and most critically, after adjusting for key confounders, co-detection was independently associated with 31% increased odds of developing SMPP, establishing it as a significant independent factor associated with this severe disease phenotype.

Our finding that co-detection independently increases the risk of SMPP aligns with a growing body of literature suggesting synergistic or additive pathogenic effects in respiratory co-detection [10, 14, 15]. Notably, SMPP, as defined by the guidelines, is a composite indicator of a more severe disease phenotype. The underlying mechanisms may involve viral damage to the respiratory epithelium, increased *M. pneumoniae* attachment and invasion, and/or dysregulation of the host immune response leading to excessive inflammation [16–18]. While co-detection was linked to greater overall severity, it did not significantly increase the rates of specific pulmonary or extrapulmonary complications.

Our stratified analysis confirms that the co-detection group is clinically heterogeneous. The finding that viral–bacterial co-detection constitutes a distinct and severe phenotype is particularly noteworthy. This aligns with the known pathophysiological synergy between viruses and bacteria. Conversely, the relatively mild course associated with single-bacterial co-detection suggests that not all co-detections equally exacerbate the severity of MPP. These insights move beyond a binary co-detection vs. no co-detection paradigm and underscore the importance of delineating the specific co-detection pattern for accurate risk stratification and potential guidance of pathogen-directed therapy.

The significantly higher hospitalization costs and increased need for bronchoscopy procedures in the co-detection group highlight substantial resource implications, which may reflect both greater disease severity and potentially more intensive diagnostic and management approaches in these patients [19]. These findings underscore the importance of early identification and aggressive management of co-infected patients, not only to improve clinical outcomes but also for optimal hospital resource allocation. However, the similar requirement for respiratory support between two groups, suggests that while co-detection prolongs illness and increases diagnostic and supportive care needs, it may not drastically increase the proportion of children who progress to the most critical level of respiratory failure requiring ventilatory support. Findings from an Italian study on the epidemiology and clinical impact of MP infection spanning from 2017 to 2024 support this conclusion: although the number of post-pandemic cases has rebounded significantly, the clinical severity of the disease, as measured by the proportion of children requiring respiratory support and admission to pediatric intensive care units, did not increase significantly before or after the pandemic [20]. Outcomes such as hospitalization costs, length of stay, and bronchoscopy rates are influenced by both the intrinsic severity of illness and institutional clinical management protocols; thus, they represent healthcare utilization rather than pure disease burden.

Our study was conducted from 2023 to 2024, a period marked by the global resurgence of *M. pneumoniae* following the relaxation of COVID-19 pandemic restrictions. This unique epidemiological background must be considered when our results are interpreted. Widespread nonpharmaceutical interventions during the pandemic likely created a substantial pool of susceptible individuals, particularly of younger children with no prior immunity, contributing to the intense epidemic wave observed during our study period [21]. This "immune debt" may have influenced both the overall incidence of MPP and the demographic profile of infected children. Furthermore, recent reports have noted increased rates of SMPP and a shift in the age distribution of infections have been published during the post-pandemic period. Although our study design cannot establish a direct causal link, the potential for altered population immunity and immune dysregulation following the pandemic is a relevant contextual factor that may have contributed to the severity patterns we observed. Finally, evolving healthcare-seeking behaviors and the increased utilization of advanced multiplex PCR diagnostics in the post-COVID-19 era may have affected hospitalization thresholds and pathogen detection sensitivity, potentially influencing the measured prevalence of co-detection.

Interestingly, older age was independently associated with SMPP in children with co-detection. This suggests distinct underlying mechanisms. Younger children, owing to immunological naivete and frequent exposure in congregate settings (e.g., daycare), may be at greater risk for concurrently acquiring multiple respiratory pathogens. In contrast, our study and others have highlighted that the host immune response to *M. pneumoniae* infection itself is profoundly age specific. Prior studies have reported that school-aged children (6–12 years) with MPP, particularly SMPP, exhibit a cytokine profile that is indicative of immune dysregulation, including significant downregulation of the JAK-STAT signaling pathway and T cell-related functions [e.g., interleukin (IL)-2 and IL-12p70 levels], along with elevated levels of markers of inflammation and apoptosis (e.g., tumor necrosis factor receptor 2 and chitinase-3-like-1) [22]. These age-specific immunologic features may predispose older children to a more dysregulated host response to primary *M. pneumoniae* infection, which could contribute to severe disease progression, independent of co-detection status.

Our study advances the field by providing a large-scale, adjusted analysis that isolates the independent effect of co-detection from other inflammatory markers. The strong, independent association of elevated D-dimer levels with SMPP reaffirms its role as a key biomarker for severe disease, potentially indicating endothelial injury and a hypercoagulable state [23]. Interestingly, the finding of slightly lower CRP levels in the co-detection group despite a higher SMPP incidence warrants further investigation. This finding may suggest that the inflammatory response during co-detection, particularly with viruses, involves pathways that are not fully captured by CRP levels or differ in their kinetics from those of pure *M. pneumoniae* infection. Future studies with broader immune profiling are needed to elucidate this phenomenon. Our study has several strengths, including the use of a large, well-characterized cohort from a major tertiary center, comprehensive pathogen detection, the use of contemporary guidelines to define SMPP, and multivariable adjustment for key confounders. However, limitations must be acknowledged. First, the single-center, retrospective design may limit generalizability, and residual confounding (e.g., by unmeasured socioeconomic or treatment factors) is possible. Second, our study focused on hospitalized children; the impact of co-detection in outpatient settings may differ. Third, detailed data on antibiotic treatment regimens (e.g., specific agent, timing of initiation, duration) were not available for comprehensive analysis. Differences in management strategies could influence disease progression and healthcare utilization, representing a potential source of residual confounding. Fourth, a key limitation is the challenge of differentiating true pathogenic co-detection from coincidental detection. Although sensitive, molecular methods cannot be used to determine the active pathogenic role of each identified microbe, particularly for viruses and for *M. pneumoniae* itself, which is known to be shed for prolonged periods [24]. Fifth, we analyzed co-detection as a binary exposure. This approach does not account for the potential heterogeneity in clinical impact among different co-detection patterns (e.g., single virus vs. viral–bacterial). The aggregated effect we reported represents an average across these patterns. Finally, our cost analysis was descriptive. Future studies employing more sophisticated economic modeling could better delineate the drivers of increased healthcare utilization associated with co-detection.

In conclusion, this study demonstrates that respiratory co-detection is present in most hospitalized children with MPP and is independently associated with progression to SMPP, a composite indicator of severe disease. Co-detection is associated with a more protracted clinical course and greater healthcare utilization. This observational study identified associations but not proven causal relationships, and the relative contribution of each detected pathogen to the disease severity remains unclear. These findings lend support to the potential clinical utility of comprehensive pathogen testing in children hospitalized with MPP. The detection of a co-detection may identify children at a higher risk of SMPP, who might therefore benefit from closer monitoring. This approach may help mitigate the prolonged morbidity and economic burden associated with co-detection.

## Data Availability

De-identified data underlying the findings reported in this manuscript can be made available from the corresponding author upon reasonable request, subject to approval by the institutional ethics committee of the Children’s Hospital of Fudan University.
